# Empathy levels among health sciences professors in Latin America: distribution, classification and gender differences

**DOI:** 10.1186/s12909-025-08224-1

**Published:** 2025-12-10

**Authors:** Víctor P. Díaz-Narváez, José Gamarra-Moncayo, Rubén Eduardo Vázquez-García, Jaime Hernández de León, Luz Marina Alonso Palacio, Margarett Cuello-Pérez, Natalia Fortich-Mesa, Luis Montero Saldaña, Laura Sánchez Jiménez, Nuvia Estrada-Méndez, Carolina More Toro, Irma Andrade Valles, Yolima Pertuz Meza, Jorge Bilbao Ramírez, María G. Silva-Vetri, Eugenia González-Díaz, Adán Alexis Acosta Martínez, Lesbia Tirado Amador, Sendy Meléndez Chávez, Juan David Salcedos Salgado, María Alicia Agudelo Giraldo, Adalberto Llinas Delgado, Jesús Alonso Cabrera, Sara Huerta-González

**Affiliations:** 1 Department of Research, Faculty of Dentistry, Universidad Andres Bello, Santiago, Chile; 2 Faculty of Medicine, Universidad Católica Santo Toribio de Mogrovejo, Chiclayo, Peru; 3 Faculty of Medicine, Universidad Veracruzana, Poza Rica, México; 4 Universidad del Norte, Health Sciences Division, Barranquilla, Colombia; 5 Faculty of Health Sciences, Corporación Universitaria Rafael Núñez, Cartagena, Colombia; 6 Centro de Investigación de Ciencias Médicas y Bioquímicas, Faculty of Medicine, Universidad Autónoma de Chiriquí, David, República de Panamá; 7 Faculty of Health Sciences, Universidad Latinoamericana de Ciencia y Tecnología, San José, Costa Rica; 8 Faculty of Dentistry, Universidad Evangélica de El Salvador, San Salvador, El Salvador; 9 Department of Nutrition and Dietetics, Faculty of Health Sciences, Universidad de Atacama, Copiapó, Chile; 10 Faculty of Health Sciences, Universidad Autónoma de Coahuila, Torreón, Coahuila México; 11 Faculty of Health Sciences, Universidad Cooperativa de Colombia, Santa Marta, Colombia; 12 Faculty of Health Sciences, Universidad Libre, Barranquilla, Colombia; 13 Faculty of Health Sciences, Universidad Nacional Pedro Henríquez Ureña, Santo Domingo, República Dominicana; 14 Faculty of Health Sciences, Universidad Central del Este, San Pedro de Macorí, República Dominicana; 15 Faculty of Health Sciences, Universidad Autónoma de Santa Ana, Santa Ana, El Salvador; 16 Faculty of Health Sciences, Universidad del Sinú, Cartagena, Colombia; 17 Faculty of Nursing, Universidad Veracruzana, Lázaro Cárdenas 801, Poza Rica, Veracruz, Morelos 93340 México; 18 Faculty of Health Sciences, Universidad del Magdalena, Santa Marta, Colombia; 19 Faculty of Educational Sciences, Universidad del Atlántico, Barranquilla, Colombia; 20 Faculty of Health Sciences, Universidad del Atlántico, Barranquilla, Colombia; 21 Basics Sciences Division, Universidad del Norte, Barranquilla, Colombia

**Keywords:** Empathy, Health sciences education, Professors

## Abstract

**Background:**

Teachers are considered potential empathetic mentors who can serve as positive role models in the development of empathy skills among students in health sciences disciplines, positively influencing their relationships with healthy or ill individuals. Objective: To estimate and classify the levels of empathy among health sciences professors in Latin America and compare their distribution by gender. Methods: This was a descriptive- analytical cross-sectional study design, involving 1,128 health sciences professors from six Latin American countries. The Jefferson Scale of Empathy – Health Professions version (JSE-HP) was administered. Descriptive statistics were applied, along with confirmatory factor analysis (using the WLSMV estimator) and analysis of variance by gender. Empathy levels were calculated based on established cut-off points.

**Results:**

More that half of the professors showed empathy levels in the medium to very low range. Measurement invariance by gender was supported. Statistically significant but small differences were observed, with women scoring slightly higher in Perspective Taking dimension and overall empathy.

**Conclusion:**

Health sciences faculty in Latin America showed predominantly moderate empathy levels with small gender differences. These findings emphasize the need to strengthen empathy support in academic settings.

**Supplementary Information:**

The online version contains supplementary material available at 10.1186/s12909-025-08224-1.

## Introduction

Empathy is a process of intersubjective connection between healthcare professionals and patients, forming the basis of effective communication in the therapeutic relationship [1, 2]. Its benefits, include greater treatment adherence, positive outcomes in the health-disease process, and increased user satisfaction with the care received [3–7].

Currently, health sciences students come into contact with patients early in their training, with this interaction increasing both quantitatively and qualitatively as they enter their clinical education stage. During this period, professors must pay close attention not only to clinically correct care but also to the appropriate attitudinal approach to patients. Which underscores the need for professors to ensure not only clinical competence but also an empathic and attitudinal approach [8, 9].

Empathy is a complex concept. Various definitions exist regarding empathy itself, its underlying components, and the very definitions of those components [10–15]. This conceptual variability may reflect the absence of a fully developed theory of empathy as a construct, and possibly the fact that empathy intersects with other attributes [15, 16] that modulate it and may act as mediators between empathy and other constructs of interest [17–21], including factors such as culture [22].

Although empathy has been conceptualized in multiple ways, it is generally understood as involving both cognitive and affective components that are influenced by individual and contextual factors. Genetic, developmental and environmental aspects contribute to its complexity, but for education purpose, empathy is best considered as a professional skill shaped by personal traits and the environment [23–27].

Empathy has been found to have heritable aspects [28, 29], adding further complexity to its assessment as a social skill. Additionally [30]. Professors play a central role in modeling and fostering empathy. Their ability to understands student´s motivations and acts role models has been associated with the development of prosocial skills and ethical practice among future healthcare professionals [31–36], However, challenges such as institutional pressures and the predominance of biomedical perspectives may inhibit the cultivation of empathy in academic settings [37–40].

These findings are important for the teaching–learning process of empathy. Since students typically enter university around age 18, they theoretically do so with a relatively consolidated emotional structure. Thus, there may be a narrow window of opportunity for emotional empathy training to have a significant impact, while the window for cognitive empathy training remains relatively broad [32, 41, 42].

Altogether, these findings suggest that teaching empathy is an extremely complex task for both professors and the institutions responsible for comprehensive student development [43, 44]. In this regard, professors must possess attributes that allow them to successfully foster empathy in students. They must be able to understand students’ internal motivations and reactions, thereby enhancing the impact of their teaching [45], promoting the development of empathy, and creating constructive learning experiences [46]. Moreover, role modeling—teaching through imitation—is one of the four main pillars of empathy development in higher medical education [47]. Research suggests that empathetic professors serve as positive role models for students in health-related disciplines and help strengthen students’ prosocial skills [48]. This modeling plays a significant role in shaping sensitive and ethical future healthcare professionals [49].

As a predictor of prosocial behavior, empathy faces important challenges. Several authors agree that the commodification of educational and healthcare systems may replace the compassionate human bond with a commodified, transactional one. In this context, empathy may become a burden, demanding excessive cognitive and emotional resources from “human capital,” leading to stress and burnout among teachers, social workers, and healthcare personnel [50, 51].

Studies conducted in Latin America has shown variability in empathy among professors and students, suggesting difficulties in its transmissions [8, 52] through traditional teaching methods.

Evidence supports the value of educational strategies based on active learning [53], collaboration and affective-experiential [54] approaches to strengthen empathy [55]. It is important to consider that the prior experiences and empathic capacities of the teacher-model must be combined with the creation of a safe, positive learning environment that enables high-quality patient care, including the exercise of empathy [56]. In light of the above, it is evident that the professor’s role is deeply involved in the development of empathy in future healthcare professionals.

Despite its importance, there is still little research on empathy among health sciences professors in Latin America.

Most previous studies on empathy in Latin America have focused primarily on medical, dental, and nursing students; therefore, the available evidence from faculty studies is limited. professors play a fundamental role as role models in the teaching- learning process, and their levels of empathy can directly influence the educational environment. Thus, examining empathy among health sciences faculty provides novel and relevant contributions to the regional literature.

Therefore, this study aimed to answer question: What are the levels and distribution of empathy among health sciences professors in Latin America? We hypothesized that empathy levels would be predominantly low to medium, with potential differences by gender. Consequently, the objective was to estimate and classify the levels of empathy among health sciences professors in Latin America and compare their distribution by gender.

## Methods

This was a descriptive- analytical cross-sectional, study. Where the levels of empathy among health sciences professors in Latin America were estimated and classified, and their distribution by gender was compared. The population studied consisted of professors who directly instruct students in Nursing, Kinesiology/Physiotherapy, Medicine, Nutrition, Dentistry, and related fields. The participating professors were from various universities in six countries: Chile, Colombia, Costa Rica, El Salvador, Mexico, and the Dominican Republic. A non-probabilistic convenience sampling method was used, including professors from the participating universities who met the inclusion criteria and were available at the time of the study.

The final sample size was *n* = 1128. Although a non-probabilistic convenience sampling strategy was applied to estimate the sample size, methodological recommendations for confirmatory factor analysis were followed, which suggest a minimum of 200 participants or 10 to 20 participants per item in psychometric studies [57, 58]. Given that the Jefferson Empathy Scale for Health Professions (JSE-HP) consists of 20 items, a minimum of 400 participants would have been sufficient. The present study exceeded these recommendations (*n* = 1128), ensuring statistical power, precision, and stable estimates in the analyses, including measurement invariance by gender.

Participants were evaluated in faculty lounges and clinical area offices where they conduct teaching, ensuring heterogeneity in terms of specialties and countries to maximize the sample’s diversity. The instruments were administered by trained facilitators who ensured the voluntary participation of the professors. The scale was administered collectively to faculty members during scheduled academic sessions, ensuring anonymity and confidentiality, and without the presence of institutional authorities. Thes conditions sought to minimize social desirability bias.

### Procedures

Professors were invited to participate through institutional means such as email, or through direct contact within their academic environments. Those who agreed to participate were informed about the study´s objectives and gave their informed consent. The questionnaire was distributed in printed form and completed individually without the presence of institutional authorities.

Participants in this study were health sciences professors who provided direct teaching in Nursing, Kinesiology/Physiotherapy, Medicine, Nutrition, Dentistry, or related programs at universities in Chile, Colombia, Costa Rica, El Salvador, Mexico, and the Dominican Republic; professors of any gender, aged 18 or older, who voluntarily agreed to participate after reading and reading and signing the informed consent form were included. Faculty members with exclusive administrative roles or those who did not complete the questionnaire were excluded.

Recruitment was carried out through institutional invitations and direct contact in academic environments. The administration of the instruments took place at all participating universities during the period of August to November 2023, following a standardized protocol in all participating countries. Completed questionnaires were reviewed for completeness and subsequently digitized into a database for analysis.

This research was approved by the Institutional Ethics Committee of Universidad Andrés Bello in Santiago, Chile, in July 2022 (Act No. 020, July 2022). All participating universities: Colombia (Corporación Universitaria Rafael Núñez, Cartagena; Universidad del Magdalena, Santa Marta; Universidad del Norte, Barranquilla; Universidad del Sinú, Cartagena; Universidad Cooperativa de Colombia, Universidad Libre, Barranquilla, and Universidad del Atlántico, Barranquilla) Chile (Universidad Andres Bello and Universidad de Atacama, Copiapó), Costa Rica (Universidad Latinoamericana de Ciencia y Tecnología, San José), El Salvador (Universidad Evangélica de El Salvador, Universidad Autónoma de Santa Ana, Santa Ana), Dominican Republic (Universidad Nacional Pedro Henríquez Ureña, Santo Domingo and Universidad Central del Este, San Pedro de Macorí) and Mexico (Universidad Veracruzana, Universidad Autónoma de Coahuila), adhered to the resolution issued by the aforementioned Ethics Committee.

The study was classified as low risk and conducted in accordance with national and international ethical standards for research with human participants (Declaration of Helsinki, 2024).

Participants were informed about the research objectives of the study, and their participation was entirely free and voluntary, as confirmed through the reading and signing of the informed consent form.

### Instrument

Empathy: The scale used was the *Jefferson Scale of Empathy–Health Professions* (JSE-HP) [59].

This scale, originally developed for health professionals and students, it has also been applied in faculty populations, where empathy plays a critical role in role-modeling and educational interactions. In this study, we administered the validated Spanish-language version of the JSE-HP, previously adapted and validated in Latin American populations [59], ensuring cultural and linguistic appropriateness for this context.

Although other empathy instruments exist, such as the Interpersonal Reactivity Index (Care Measure), the JSE-HP was selected because it specifically operationalizes clinical empathy within health professions education [59, 60]. This version has been previously adapted and applied in Latin America populations, demonstrating adequate psychometric properties. Studies conducted in Chile [8, 52], have reported adequate reliability and factorial validity. Thes studies support the use of the JSE-HP in Spanish in the present research context.

The scale has demonstrated adequate internal consistency (α = 0.78–0.92) and cultural validity (α >0.80). It also shows appropriate correlations with other psychological constructs [58, 59].

This instrument was used under license from the Asano-Gonnella Center for Research in Medical Education and Health Care, Thomas Jefferson University (Order ID: 10905). The scale comprises 20 items designed to assess empathy levels toward patients among health sciences students across various specialties. Items are rated on a 7-point Likert scale ranging from 1 (Strongly Disagree) to 7 (Strongly Agree). The JSE-HP measures three dimensions: Compassionate Care (CC) (Items 1, 7, 8, 11, 12, 14, 18, 19), Perspective Adoption (PA) (Items 2, 4, 5, 9, 10, 13, 15, 16, 17, 20), and Walking in the Patient’s Shoes (WIPS) (Items 3 and 6).

### Data analysis

Descriptive statistics were used to assess univariate normality (skewness and kurtosis) and multivariate normality (Mardia’s test) of the instrument. Confirmatory Factor Analysis (CFA) was conducted using the WLSMV estimator, which is recommended when working with large datasets (*n* >1000) and violations of normality are present, as it provides less biased estimates [57]. Model fit was evaluated using the following criteria: CFI >0.90, TLI >0.90, RMSEA < 0.08, and SRMR < 0.05 [61]. Reliability was assessed using the omega coefficient, with values above 0.70 considered acceptable [61]. Invariance by gender was also tested, using the following thresholds: ΔCFI < 0.01 and ΔRMSEA < 0.015 [62]. Since groups will be compared and this is an essential feature that a measuring instrument must have. Cut-off points were established to define five levels of the scale [63].

All analyses were performed using JASP and R with the RStudio interface, employing the following packages: *lavaan version* 0.6–19, *psych version* 2.4.12, *semTools version* 0.5–6.5, and *MVN version* 5.9. A comparative analysis by sex was performed using the nonparametric Mann-Whitney U test, since violations of normality were found. Likewise, the effect size (biserial correlation) was considered.The dataset for this study is available in the following public repository: https://osf.io/q2u9d?view_only=ee848d1f02764f239d40386dc3790028.

## Results

All 1,128 professors who participated in the study completed the survey correctly, resulting in a 100% response rate for properly completed instruments. The sample included participants from seven Latin American countries, with variability in subsample sizes; some countries contributed large groups while others provided smaller subsamples.

The average age of participants was 43.63 years (SD = 11.61). The sociodemographic characteristics of the sample are presented in Table 1.

**Table 1:** Sociodemographic characteristics of the professors

Data	Characteristics	*n* (%)
Gender	Masculine	396 (35.11)
	Femenine	732 (64.89)
Country	Chile	272 (24.11)
	Colombia	508 (45.04)
	Costa Rica	27 (2.39)
	El Salvador	100 (8.87)
	Mexico	17 (1.51)
	Panama	55 (4.88)
	Dominican Republic	149 (13.2)
Speciality	Basic Sciences	46 (4.08)
	Social Sciences	11 (0.98)
	Nursing	159 (14.1)
	Kinesiology and Physiotherapy	69 (6.12)
	Medicine	291 (25.8)
	Nutrition	23 (2.03)
	Dentistry	524 (46.45)
	Others	5 (0.44)

Note: Frequency (n) and percentages (%); gender was self-reported

### Descriptive analysis of the items

Table 2 shows that some items [2, 4], and [20] exhibited high kurtosis values, which are considered indicators of severe non-normality [63]. Additionally, multivariate normality was assessed using Mardia’s test, and the results indicated that this assumption was not met (*p* <.001).

**Table 2:** Univariate descriptive statistics of the items

Items	M	σ	g1	g2	Items	M	Σ	g1	g2
Empathy scale									
1	4.37	2.42	−0.21	−1.60	11	5.77	1.78	−1.44	0.86
2	6.49	1.31	−3.15	9.45	12	5.48	2.10	−1.20	−0.11
3	5.52	1.81	−1.16	0.15	13	6.03	1.54	−2.05	3.61
4	6.53	1.03	−3.13	11.17	14	6.05	1.70	−1.93	2.52
5	5.96	1.49	−1.78	2.74	15	6.17	1.38	−2.06	4.01
6	5.46	1.84	−1.14	0.09	16	6.26	1.29	−2.38	5.88
7	5.95	1.70	−1.71	1.79	17	5.99	1.39	−1.83	3.21
8	4.95	2.25	−0.66	−1.14	18	3.58	1.96	0.29	−1.09
9	6.21	1.38	−2.23	4.66	19	6.01	1.68	−1.80	2.12
10	6.16	1.36	−2.14	4.66	20	6.49	1.21	−3.23	10.75

Note. M = Mean, σ = Standard deviation, g₁ = Skewness, g₂ = Kurtosis

### Evidence of internal structure validity and reliability

**Fig. 1:**
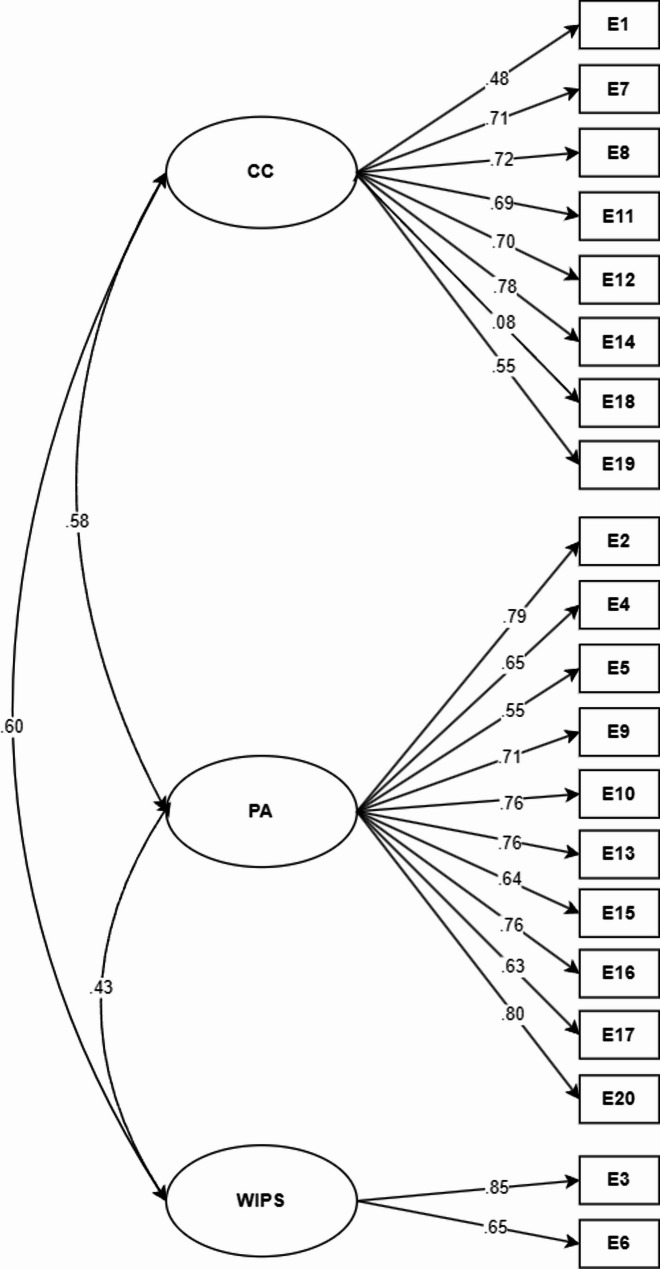
Oblique three-factor model of empathy. Note: CC = Compassionate Care, PA = Perspective adoption, WIPS = Walking in the patient’s shoes, EMP = Empathy

The original model of three oblique factors was tested, yielding acceptable fit indices: χ² (df) = 913.048 (167); CFI = 0.94; TLI = 0.93; RMSEA [90% CI] = 0.063 [0.059–0.067], SRMR = 0.059. Although these indices are within commonly accepted thresholds, they should be interpreted as adequate but modest indicators of model fit (Fig. 1).

Despite the adequate structural performance, item 18 showed a factor loading considerably lower than expected; however, it was retained to preserve the original composition of the instrument, noting that is weaker performance may slightly reduce the reliability of the corresponding subscale.

Regarding reliability, McDonald´s omega coefficients (ω) were calculated as follows: ω = 0.74 for Compassionate Care, ω = 0.87 for Perspective Adoption, and ω = 0.67 for Walking in the Patient’s Shoes [64]. The latter value falls below the threshold generally considered acceptable and should therefore be interpreted with caution.

### Measurement invariance by gender

Because valid group comparisons require that the construct be measured equivalently across groups, we tested measurement invariance by gender before analyzing differences. The results supported configural, metric, scalar and strict invariance, with ΔCFI ≤ 0.01 and ΔRMSEA ≤ 0.015 across successive models. These findings indicate that the factor structure of the JSE-HP is invariant across male and female faculty members, supporting the validity of gender- based comparisons.

Table 3 shows that the empathy scale demonstrated strict measurement invariance by gender. This verification is necessary because without evidence of invariance, it is not possible to ensure that men and women interpret the items in the same way or that the scores reflect the same construct in both groups. In the absence of invariance, the differences observed could be due to measurement biases rather than actual differences in the levels of latent variable.

**Table 3:** Comparison of invariance levels for the empathy scale

Level	X^2^ (gl)	*P*	CFI	ΔCFI	RMSEA	ΔRMSEA
Configural	1170.58 (334)	< 0.001	0.93		0.07	
Metric	990.84 (351)	< 0.001	0.94	0.01	0.06	−0.01
Scalar	1086.58 (448)	< 0.001	0.94	0.00	0.05	−0.01
Strict	1086.58 (448)	< 0.001	0.94	0.00	0.05	0.00

Note: ΔCFI and ΔRMSEA represent changes between consecutive models. Evidence of measurement invariance is considered when ΔCFI ≤ 0.01 and ΔRMSEA ≤ 0.015 (Cheung & Rensvold, 2002; Chen, 2007). The results indicate configural, metric, scalar and strict invariance, supporting the validity of gender-based comparisons. df = degrees of freedom; p = level of significance

*CFI* Comparative fit index, *RMSEA* Root mean square error of approximation

### Cut-off points for the empathy scales

Empathy levels were categorized into five groups: very high, high, average, low and very low. The cut-off points for these categories were established, based on recommendations from previous literature [57] that analysed this scale.

Thresholds were derived from percentile-based distributions, where scores below the 25th percentile were classified as low or very low, between the 25th and 75th percentile as average, and above the 75th percentile as high or very high. This procedure has been consistently applied in Latin American validations of the JSE-HP, ensuring comparability across populations [60].

Table 4 shows the cut-off points for the dimensions of empathy, based on five levels and their corresponding minimum and maximum scores for each dimension.

**Table 4:** Cut-off points for the dimensions of the empathy scale

	CC	AP	CZP	EMP
Very high	54	70	14	135
High	50	69	13	125
Average	44	64	12	118
Low	37	59	9	108
Very low	24	43	5	82
Min-Max	11–56	10–70	2–14	26–139

Note. *CC* Compassionate care, *PA* Perspective adoption, *WIPS* Walking in the patient’s shoes, *EMP* Empathy

### Empathy levels of the participating professors

Table 5 shows that professors with medium, low, and very low levels account for 54.52% in the CC dimension, 51.3% in the PA dimension, 53.19% in the WIPS dimension, and 50.26% in overall empathy.

**Table 5:** Empathy levels of the professors (*n* = 1128)

Levels	CC		AP		CZP		EMP	
	*n*	%	*n*	%	*n*	%	*n*	%
Very high	206	18.26	202	17.91	405	35.90	342	30.32
High	307	27.22	350	31.02	123	10.91	219	19.42
Average	318	28.19	276	24.47	273	24.20	274	24.29
Low	238	21.10	239	21.19	245	21.72	236	20.92
Very low	59	5.23	61	5.41	82	7.27	57	5.05

Note. *CC* Compassionate care, *PA* Perspective adoption, *WIPS* Walking in the patient’s shoes, *EMP* Empathy

Table 6 presents the results of the descriptive statistics estimates for empathy and its dimensions among professors of both genders.

**Table 6:** Descriptive measures of empathy and its dimensions among male and female professors

Dimensions	Group	*n*	Mean	95% CI	SD
Compassionate care	Total sample	1128	42.16	[41.62–42.70]	9.26
	Male professors	396	42	[41.13–42.86]	8.86
	Female professors	732	42.25	[41.56–42.94]	9.47
Perspective Adoption	Total sample	1128	62.29	[61.79–62.79]	8.57
	Male professors	396	61.18	[60.35–62.00]	8.33
	Female professors	732	62.89	[62.26–63.52]	8.65
Walking in the Patient’s Shoes	Total sample	1128	10.99	[10.81–11.17]	3.11
	Male professors	396	10.82	[10.53–11.10]	2.89
	Female professors	732	11.08	[10.85–11.31]	3.22
Empathy	Total sample	1128	115.44	[114.49–116.39]	16.22
	Male professors	396	113.99	[112.45–115.54]	15.63
	Female professors	732	116.22	[115.02–117.42]	16.49

Note: n = sample size; gender was self-reported

*SD* Standard deviation

With regard to the analysis of differences according to gender, it was found that they were significant for the dimensions of perspective adoption, walking in the patient’s shoes, and empathy in general, with a slight inclination toward women. However, at a practical level, the effect sizes were small in all cases. These comparisons are summarized in Table 7, which presents the differences in empathy and its dimensions between male and female professors.

**Table 7:** Comparison of empathy and its dimensions between male and female professors

Dimensions	Groups	S-W	L-T	U	Median	*p*	*r*_bis_
CC	Male	< 0.001	0.175	149,111	44	0.423	0.029
	Female				44		
PA	Male	< 0.001	0.677	170,415	63	< 0.001	0.176
	Female				65		
WIPS	Male	< 0.001	0.019	157,741	12	0.013	0.088
	Female				12		
EMP	Male	< 0.001	0.386	161,354	116	0.002	0.113
	Female				120		

Note: S-W: Shapiro-Wilk (normality test); L-T: Levene’s Test (homoscedasticity test); rbis: Biserial correlation (effect size)

## Discussion

It is relatively common to estimate empathy levels using the Jefferson Scale of Empathy (JSE) in any of its versions [65–69] without conducting psychometric validation of the instrument. Given that empathy is a construct with latent dimensions, self-administered instruments are generally known to be influenced by various factors that may alter the internal structure of the original model and introduce uncontrolled biases [60, 69–71]. In this study, the construct validity, reliability of the data, and measurement invariance across gender were confirmed. Empathy levels among professors were predominantly moderate, with a considerable proportion classified as low or very low. These findings should not be interpreted as insufficient, but rather as descriptive indicators of the distribution of empathy in this sample, consistent with results previously reported in studies of health sciences faculty [60, 71].

Whether empathy is higher in women than in men remains a debated issue; empirical studies on this matter yield contradictory results [72–74]. Gender differences were statistically significant, favouring women; however, the size of these effects was small and of little practical relevance. Suggesting that gender should not be overestimated when interpreting empathy levels. Regarding item 18, its lower factor loading is consistent with the findings of previous studies, where similar difficulties have been documented [75–77]. This reinforces the need for ongoing evaluation of this item in different cultural contexts.

The analysis of the percentage distribution of empathy levels and their dimensions (Table 5) is also complex, given that the full meaning and impact of a professor’s empathy level on the development of student empathy are not yet fully understood [47]. Previous studies in Latin America [52, 72, 78] have reported heterogeneous results regarding empathy levels in faculty and students. Our findings contribute to this body of evidence and emphasize the need for more research in the region to better understand contextual and cultural influences on the development of empathy among health sciences educators.

The hypothesis that there is a positive correlation between professors’ and students’ empathy levels seems logically sound. The findings of this study show that over 50% of professors scored in the low, very low, or medium empathy range (with medium considered insufficient, based on the premise that professors should ideally have high or very high empathy levels). This comparison should be interpreted cautiously, since contextual differences among countries and disciplines may partly explain the variability in findings.

Considering this, and the evidence that empathy can be effectively taught [32, 52, 79, 80], an important question arises: What is the impact on health sciences students’ development of empathy when more than half of their professors demonstrate medium, low, or very low levels of empathy and its dimensions? This question cannot be answered by the current study and requires further research. This issue has not been adequately addressed in similar research, despite noteworthy empirical findings [79–82]. However, our data suggest that teachers’ empathy may be influenced not only by also by institutional and cultural contexts.

Some recent studies have found high levels of empathy among both students and professors [79], highlighting the importance of assessing this competency in both groups. However, they have not analyzed the broader significance of this finding. Weissman [82] emphasizes the need to measure empathy in faculty members to understand their influence on students’ empathic development. Similarly, a systematic [83] suggests that a possible cause of empathy decline in students may be negative role models or even mistreatment by mentors. A coherent theoretical framework is needed to correctly interpret empirical findings, such as those presented here—and to answer key questions like the one posed above. However, such a unifying theory is still lacking.

The educational modeling of empathy may find theoretical support in Albert Bandura’s Social Learning Theory, which posits that humans learn through observation, imitation, and modeling [84]. Nevertheless, the current state of the literature does not allow us to attribute empathy decline solely to professors, since empathy is shaped by biological, environmental, cultural, and institutional factors, among others [80, 84–87].

This multiplicity of influences may help contextualize the variability observed in empathic behavior. For instance, studies in North American and European populations have documented a progressive decline in empathy levels as students advance in their health programs [80]. In contrast, research in Latin American populations has shown fluctuating results that do not fully align with the widely described “decline” model. Such variability has been observed in studies with dental [85] and nursing students [87, 88], while other reports have suggested differences by gender particularly in medical programs [88]. These findings illustrate the complexity of empathy development and highlight the need for cautious interpretation across different contexts.

The findings from these studies may be partly interpreted in light of the “influence” that professors’ varying empathy levels could have on their students [85]. This perspective is consistent with mechanism of social learning and modeling as potential pathways for behavioural transmission. However, these considerations remain hypothetical, since our data do not directly evaluate such processes. Important questions remain about the factors underlying empathy variability in professors and how these may affect the teaching- learning dynamic. This is particularly relevant given that today´s health sciences professors were once students trained by faculty who themselves may have exhibited heterogeneous empathy levels, which could have influenced their own empathic development.

Another hypothesis that may help explain our findings is that the emphasis on evidence-based practices has contributed to the erosion of a holistic approach in the patient–provider relationship [89–91]. Contributing factors may include the high number of patients in healthcare systems, time pressure during consultations, a predominantly curative approach (especially in medical programs), and insufficient empathy training [89].

Moudatsou et al. [90] note that traits such as superiority complexes, presumptuousness, fear of crossing boundaries, lack of self-awareness, and anxiety are often seen among healthcare professionals—and may also be present in faculty at health sciences schools in Latin America. These traits suppress the expression of empathy and may help explain the results observed in this study. This supports the idea that, although professor modeling is important and measuring empathy is essential, we must also address structural shortcomings in both healthcare and educational systems that impede effective empathy training [49, 50].

These systemic issues appear to be associated with dispositional attitudes shaped by phylogenetic and ontogenetic development [24, 26]. The empathy expressed by professors—partly influenced by their own past experiences [56]—may contribute to the type of environment they create for teaching and learning, ideally fostering a safe space for the development of health knowledge and professional skills such as empathy. As highlighted by several authors [8, 15, 32, 41, 52, 60, 71–73, 81, 83, 84], the teaching–learning process is inherently complex requiring careful preliminary assessment to design responsible and effective strategies for enhancing empathy in students. Comparable findings have also been reported in international studies, which emphasize that contextual, cultural, and institutional conditions significantly shape how empathy is expressed and transmitted in academic settings.

The pedagogical component of empathy must go beyond curriculum content and engage, in practice, with ontogenetic and sociogenetic factors. Paulo Freire’s educational philosophy [92], which emphasizes dialogical and liberating education aimed at social awareness and transformation, aligns well with this approach. It seeks to prevent health professionals from becoming passive or commodified within the logic of market-driven education and healthcare systems [50], thereby reducing patient relationships to transactional interactions.

Given the complexity of the teaching–learning process as described, Davidson and Begley [93] have argued that advances in neuroplasticity suggest the possibility of modifying neural patterns through targeted training. Such processes may help counterbalance genetic predispositions and prior developmental experiences, thereby contributing to empathy development. This perspective supports the idea that empathy is not solely an innate trait but can be intentionally fostered, with potential implications for emotional education [93, 94]. Nevertheless, it remains uncertain to what extent neuroplasticity—typically restorative—can modify the neural networks related to empathy (e.g., limbic and orbitofrontal systems), which begin forming very early in life [23, 24].

These interpretations should be as hypothetical explanations rather than direct conclusions. While theoretical frameworks and international evidence suggest that systemic, neurobiological, and pedagogical factors may influence empathy, our cross-sectional data do not allow causal inferences. Future studies will be needed to examine these possibilities in more depth.

Theories like those presented here offer valuable perspectives for addressing empathy in education, especially through transformative pedagogy that targets not only students but also faculty and the educational system itself. This approach requires institutional models that shift away from purely technical-procedural frameworks toward more comprehensive, humanistic perspectives.

It is therefore essential to promote curricula that incorporate empathy-related social-emotional and cognitive competencies in a transversal and longitudinal manner, using active learning methods. It is also crucial to reduce academic overload, relieve productivity pressures imposed by healthcare systems, and foster more humanistic educational environments. Under these conditions, educators can build authentic, transformative pedagogical relationships, while students—through exposure to meaningful and empathic environments—can develop the sensitivity needed to replicate such relationships in their future professional practice, contributing to more humane and ethically grounded healthcare.

However, the lack of concrete empathy training strategies appears to be a widespread issue in health sciences education across Latin America. Changing this inertia is challenging but not impossible [32].

Including student perspectives could be vital in designing these strategies. A systematic metasynthesis [95] found that students often perceive a lack of conceptual clarity around empathy, leading them to question its relevance. They also believe that clinical professors should play a stronger role in empathy education and that the prioritization of clinical data over interpersonal knowledge negatively affects empathy development. Our findings may inform educational strategies, although direct recommendations should be made cautiously and require further confirmatory studies.

Meanwhile, interventions assessing empathy before and after implementation [96, 97], including quasi-experimental [98] and problem-based simulation studies [99], have reported short-term improvements in study groups. However, since these interventions were conduced over relatively brief periods within longer academic programs, it remains uncertain whether the observed gains were sustained or translated into enduring behavioral patterns. Our cross-sectional results, showing predominantly moderate empathy levels and small gender differences among faculty, are consistent with the notion that longer term developmental contextual influences may play a relevant role, although this hypothesis requires confirmation through longitudinal and intervention-based research.

In summary, developing and sustaining empathy is a long, complex process that appears to begin early in life. Within this trajectory, the university may represent an important stage for intentionally fostering empathy in students. Theories of teaching and learning converge on several premises: that empathy training should be transversal and longitudinal, embedded in active learning, and ideally integrated into the hidden curriculum. While these principles are well established at the theoretical level, there remains limited conceptual clarity about empathy—and about how to effective actions within higher education, which continues to represent a significant challenge.

The role of university professors as influential agents in the teaching-learning process suggests that their levels of empathy are highly relevant to students´ professional development. In this sense, the observation of moderate and, in some cases, low levels of empathy among Latin American professors highlights a potential challenge for academic institutions, underscoring the importance of incorporating strategies than strengthen empathy in teacher development and educational practices.

One limitation of this study is that it was not possible to establish measurement invariance across countries. This means that direct comparisons of resilience and empathy levels between national contexts should be interpreted with caution, given that the differences observed could be due, in part, to variations in the way the items are understood and answered in each country, and not solely to substantive differences in the constructs evaluated.

## Conclusion

The findings of this study regarding the empathy levels of professors in Latin America across different medical specialties reveal predominantly moderate levels, small gender differences, and variability between subscales. These results enrich the regional literature and underscore the importance of fostering empathy in academic contexts. Future research with representative and longitudinal designs is needed to confirm and extend these findings, as well as to better understand the institutional and cultural factors that shape empathy in health sciences education.

## Supplementary Information

Supplementary Material 1.

## Data Availability

The data supporting this study can be accessed through the OSF repository at the following link: [https://osf.io/q2u9d?view_only=ee848d1f02764f239d40386dc3790028](https://osf.io/q2u9d?view_only=ee848d1f02764f239d40386dc3790028).

## Abbreviations

JSE-HP: Jefferson Scale of Empathy- Health Professions version
CC: Compassionate Care
PA: Perspective Adoption
WIPS: Walking in the Patient´s Shoes
CFI: Comparative Fit Index
TLI: Tucker-Lewis Index
RMSEA: Root Mean Square Residual
OSF: Open Science Framework
df: Degrees of freedom
SD: Standard Deviation
AP: Perspective Adoption
WIPS: Walking in the Patient’s Shoes
TES: Total Empathy Score
ΔCFI: Change in the Comparative Fit Index
ΔRMSEA: Change in the Mean Square Error of Approximation
