# Supplementary material for: Empathy levels among health sciences professors in Latin America: distribution, classification and gender differences
Source: BMC Med Educ. 2025 Dec 10;26:73. doi: 10.1186/s12909-025-08224-1 (PMC12801935; doi:10.1186/s12909-025-08224-1)
Supplement: Supplementary file 1 — Supplementary Material 1. [file 12909_2025_8224_MOESM1_ESM.docx]

**Distribution and Classification of Empathy Levels in Health Sciences Professors in Latin America**

Empathic Profile of Health Sciences Professors in Latin America

Autors:

Víctor P. Díaz-Narváez^1^, Jose Gamarra-Moncayo^2^, Rubén Eduardo Vazquez-Garcia^3^, Jaime Hernández de León^4^, Luz Marina Alonso Palacio^5^, Margarett Cuello-Pérez^6^, Natalia Fortich-Mesa^7^, Luis Montero Saldaña^8^, Laura Sánchez Jiménez^9^, Nuvia Estrada-Méndez^10^, Carolina More Toro^11^, Irma Andrade Valles^12^, Yolima Pertuz Meza^13^, Jorge Bilbao Ramírez^14^, María G. Silva-Vetri^15^, Eugenia González-Díaz^16^, Adán Alexis Acosta Martínez^17^, Lesbia Tirado Amador^18^, Sendy Meléndez Chávez^19^, Juan David Salcedos Salgado^20^, María Alicia Agudelo Giraldo^21^, Adalberto Llinas Delgado ^22^, Jesús Alonso Cabrera^23^, Sara Huerta-González^24^*.

1. Research Professor of Department of Research, Faculty of Dentistry. Universidad Andres Bello, Santiago, Chile. ORCID: https://orcid.org/0000-0002-5486-0415 email: [victor.diaz@unab.cl](mailto:victor.diaz@unab.cl)

2. Research Professor. Faculty of Medicine, Universidad Católica Santo Toribio de Mogrovejo, Chiclayo, Peru. ORCID: https://orcid.org/0000-0002-0781-3616 Email: [gamarramoncayoj@gmail.com](mailto:gamarramoncayoj@gmail.com)

3. Research Professor of Faculty of Medicine. Universidad Veracruzana, Poza Rica, México. ORCID: <https://orcid.org/0009-0009-7921-4610> Email: [rubenvazquez@uv.mx](mailto:rubenvazquez@uv.mx)

4. Research Professor of Faculty of Medicine. Universidad Veracruzana, Poza Rica, México. ORCID: <https://orcid.org/0009-0009-7921-4610> Email: [jaimhernandez@uv.mx](mailto:jaimhernandez@uv.mx)

5. Research Professor of Universidad del Norte. Health Sciences Division. Barranquilla, Colombia. ORCID: https://orcid.org/0000-0002-7935-8042 email: [lmalonso@uninorte.edu.co](mailto:lmalonso@uninorte.edu.co)

6. Research Professor of Faculty of Health Sciences, Corporación Universitaria Rafael Núñez, Cartagena, Colombia. ORCID: <https://orcid.org/0000-0002-3741-3170> Email: [margarett.cuello@curnvirtual.edu.co](mailto:margarett.cuello@curnvirtual.edu.co)

7. Research Professor of Faculty of Health Sciences, Corporación Universitaria Rafael Núñez, Cartagena, Bolívar Colombia. ORCID: <https://orcid.org/0000-0003-2929-0278> Email: [natalia.fortich@curnvirtual.edu.co](mailto:natalia.fortich@curnvirtual.edu.co)

8. Research Professor of the Centro de Investigación de Ciencias Médicas y Bioquímicas, Faculty of Medicine, Universidad Autónoma de Chiriquí, David, República de Panamá. ORCID: <https://orcid.org/0009-0004-2606-0789> Email: [luis.montero@unachi.ac.pa](mailto:luis.montero@unachi.ac.pa)

9. Research Professor of Faculty of Health Sciences, Universidad Latinoamericana de Ciencia y Tecnología, San José, Costa Rica. ORCID: <https://orcid.org/0000-0003-4693-8427> Email: [sanchezj821@ulacit.ed.cr](mailto:sanchezj821@ulacit.ed.cr)

10. Research Professor of Faculty of Dentistry, Universidad Evangélica de El Salvador, San Salvador, El Salvador. ORCID: <https://orcid.org/0000-0003-2314-3015> Email: [nuvia.estrada@uees.edu.sv](mailto:nuvia.estrada@uees.edu.sv)

11. Professor Department of Nutrition and Dietetics, Faculty of Health Sciences. Universidad de Atacama, Copiapó, Chile. Email: carolina.more@uda.cl ORCID: <https://orcid.org/0000-0002-8433-0554>

12. Research Professor of Faculty of Health Sciences, Universidad Autónoma de Coahuila. Torreón, Coahuila, México. ORCID: https://orcid.org/0000-0001-8461-1033 Email: [irmaandradevalles@uadec.edu.mx](mailto:irmaandradevalles@uadec.edu.mx).

13. Research Professor of Faculty of Health Sciences, Universidad Cooperativa de Colombia, Santa Marta, Colombia. ORCID: <https://orcid.org/0000-0001-6928-4249> E-mail: [yolima.pertuz@campusucc.edu.co](mailto:yolima.pertuz@campusucc.edu.co)

14. Research Professor of Faculty of Health Sciences. Universidad Libre, Barranquilla, Colombia. ORCID: <https://orcid.org/0000-0001-7738-0740> Email: [jbilbao55@hotmail.com](mailto:jbilbao55@hotmail.com)

15. Research Professor of Faculty of Health Sciences. Universidad Nacional Pedro Henríquez Ureña, Santo Domingo, República Dominicana. ORCID: <https://orcid.org/0000-0003-2880-5778> Email: [gsilva@unphu.edu.do](mailto:gsilva@unphu.edu.do)

16. Research Professor of Faculty of Health Sciences. Universidad Central del Este, San Pedro de Macorí, República Dominicana. ORCID: <https://orcid.org/0000-0002-0992-3229> Email: [egonzalez@uce.edu.do](mailto:egonzalez@uce.edu.do)

17. Research Professor of Faculty of Health Sciences. Universidad Autónoma de Santa Ana, Santa Ana, El Salvador. ORCID:<https://orcid.org/0000-0001-6378-5739> Email: [investigador2@unasa.edu.sv](mailto:investigador2@unasa.edu.sv)

18. Research Professor of Faculty of Health Sciences. Universidad del Sinú, Cartagena, Colombia. ORCID: https://orcid.org/0000-0003-2821-6762. Email: [coordpractodontologia@unisinucartagena.edu.co](mailto:coordpractodontologia@unisinucartagena.edu.co)

19. Research Professor of Faculty of Nursing. Universidad Veracruzana, Poza Rica, México. ORCID: <https://orcid.org/0000-0002-7378-4737> Email: [smelendez@uv.mx](mailto:smelendez@uv.mx)

20. Research Professor of Faculty of Health Sciences. Universidad del Magdalena, Santa Marta, Colombia. Email: [jsalcedos@unimagdalena.edu.co](mailto:jsalcedos@unimagdalena.edu.co) ORCID: <https://orcid.org/0000-0002-6813-3948>

21. Research Professor of Faculty of Educational Sciences. Universidad del Atlántico, Barranquilla, Colombia. Email: [mariaagudelo@mail.uniatlantico.edu.co](mailto:mariaagudelo@mail.uniatlantico.edu.co) ORCID: <https://orcid.org/0000-0001-6483-1231>

22. Research Professor of Faculty of Health Sciences. Universidad del Atlántico, Barranquilla, Colombia. Email: adalbertollinas@mail.uniatlantico.edu.co ORCID: 0000-0003-0665-8180

23. Research Professor of Basics Sciences Division. Universidad del Norte, Barranquilla, Colombia. ORCID:https://orcid.org/0000-0002-2372-6678 Email: jcabrera@uninorte.edu.co

24. Research Professor of Faculty of Nursing. Universidad Veracruzana, Poza Rica, México. Email: [sahuerta@uv.mx](mailto:sahuerta@uv.mx) ORCID: <https://orcid.org/0000-0003-4276-1038>

**Corresponding author**

*Sara Huerta González. PhD. Program in Nursing, Faculty of Nursing. Universidad Veracruzana, México. Lázaro Cárdenas 801, Morelos, Poza Rica, Veracruz, México, Postal Code: 93340. Phone +52 (782) 8245700-01 extension: 43120. ORCID https://orcid.org/0000-0003-4276-1038. Institutional email: sahuerta@uv.mx Personal: [saryhuerta_2007@hotmail.com](mailto:saryhuerta_2007@hotmail.com)
